# Genome-wide analysis of brain and gonad transcripts reveals changes of key sex reversal-related genes expression and signaling pathways in three stages of *Monopterus albus*

**DOI:** 10.1371/journal.pone.0173974

**Published:** 2017-03-20

**Authors:** Wei Chi, Yu Gao, Qing Hu, Wei Guo, Dapeng Li

**Affiliations:** 1 College of Fisheries, Huazhong Agricultural University, Wuhan, China; 2 Freshwater Aquaculture Collaborative Innovation Center of Hubei Province, Wuhan, China; University of Hyderabad, INDIA

## Abstract

**Background:**

The natural sex reversal severely affects the sex ratio and thus decreases the productivity of the rice field eel (*Monopterus albus*). How to understand and manipulate this process is one of the major issues for the rice field eel stocking. So far the genomics and transcriptomics data available for this species are still scarce. Here we provide a comprehensive study of transcriptomes of brain and gonad tissue in three sex stages (female, intersex and male) from the rice field eel to investigate changes in transcriptional level during the sex reversal process.

**Results:**

Approximately 195 thousand unigenes were generated and over 44.4 thousand were functionally annotated. Comparative study between stages provided multiple differentially expressed genes in brain and gonad tissue. Overall 4668 genes were found to be of unequal abundance between gonad tissues, far more than that of the brain tissues (59 genes). These genes were enriched in several different signaling pathways. A number of 231 genes were found with different levels in gonad in each stage, with several reproduction-related genes included. A total of 19 candidate genes that could be most related to sex reversal were screened out, part of these genes’ expression patterns were validated by RT-qPCR. The expression of *spef2*, *maats1*, *spag6* and *dmc1* were abundant in testis, but was barely detected in females, while the *17β-hsd12*, *zpsbp3*, *gal3* and *foxn5* were only expressed in ovary.

**Conclusion:**

This study investigated the complexity of brain and gonad transcriptomes in three sex stages of the rice field eel. Integrated analysis of different gene expression and changes in signaling pathways, such as PI3K-Akt pathway, provided crucial data for further study of sex transformation mechanisms.

## Background

Sexual reproduction is one of the fundamental and crucial biological processes common among eukaryotes. Although most animals occur in two morphological distinct sexes, sex determination can be various and flexible. Primary sex-determining signals can either be genetically controlled or environmentally induced, or both, in some occasions [1, 2]. In animals with genetic sex determination (GSD), the primary sex of an individual is determined once the fusion of spermotozoa and egg is done. Sex-linked genes initiate sexually dimorphic pathways during development, directing the gonad to differentiate into the testis or ovary. In mammals, for example, male sex determination is controlled by the sex-determining region on Y chromosome (*SRY*), which encodes a DNA-binding protein that acts dominantly to trigger differentiation of the testes from undifferentiated gonads that otherwise develop as ovaries [3, 4]. In birds and reptiles, an SRY-related gene *sox9* is thought to dominantly control male differentiation [5–7]. Gene *dmrt1* is demonstrated to be required for testis determination in chicken by means of knockdown via RNA interference, which resulted in feminization of the embryonic gonads in genetically male (ZZ) embryos [8]. Another gene termed *dm-w*, the binding domain of which shows a strikingly high identity with that of *dmrt1*, is proved playing a crucial role in primary ovary formation of amphibians [9]. In medaka, a copy of *dmrt1* gene, named *dmrt1y*, was found expressed during male embryonic and larval development and in the Sertoli cells of the adult testes, making it responsible for male medaka sex determination [10]. The *dmy* gene was also found crucial for male medaka sexsual differentiation, mutations on *dmy* could result in all XY female offspring [11]. In animals with environmental sex determination (ESD), since lacking any consistent heritable genetic difference between the sexes, their sexual identity is established by one or several environmental cues after fertilization [12]. Environmental cues involved in ESD can be biotic or abiotic, including population density, steroids, pathogens, temperature and pH [13–15]. Temperature is most broadly studied environmental cue, particularly in reptiles [15, 16].

Generally, once the sex of an individual is determined, it will be fixed for life. But in some fish species, sex can be changed under certain circumstances, such as, being exposed to hormones. As a result of aquaculture and physiology research, to date the sex-reversing effects of a variety of chemicals have been tested on hundreds of fish species from a range of families [17–19]. However, in some particular fishes, the primary sex can be altered naturally during development, changing into the opposite sex with the genotype remained. The rice field eel, *Monopterus albus*, taxonomically belongs to teleosts, the family Synbranchidae of the order Synbranchiformes, is an important economic freshwater fish of Southeast Asia. Early study has confirmed that the rice-field eel experiences natural sex reversal during its life [20], which is that it starts its sexual life history as a female, undergoes an intersex stage, and then develops into the final male stage [21, 22]. The molecular mechanism underlies this special biological process draws many researchers’ attention, and studies showed that several sex differentiation and maturation related genes were involved. Two homologous genes, *sox9* and *sox17*, were identified in the rice field eel, which showed similar structures and high identities comparing to mammals, indicating evolutionarily conserved roles in sex determination and differentiation [23], and this speculation was supported by subsequent experimental analysis [24, 25]. The c-Jun N-terminal kinases (JNKs), a member from the mitogen-activated protein kinase family, displayed a distinct expression pattern, with a high expression level in ovary, then kept dropping down during the sex transformation [26]. It’s been known to us that androgen signaling is essential for male sexual differentiation. Study in the rice field eel androgen receptor (AR) reflected an increasing tendency on expression during gonadal transformation from ovary via ovotestis to testis, and a novel nuclear export pathway for AR has been found [27]. Two genes that encode cytochrome P450 aromatase and cytochrome P45011β-hydroxylase are found playing crucial roles during gonadal sex change, with the P450arom predominantly expressed in ovary and P45011β mainly expressed in testis [28].

The natural sex reversal severely affects the sex ratio and thus decreases the productivity of the rice field eel. How to manipulate this process is one of the major issues for the rice field eel stocking. So far the genomics and transcriptomics data available for this species are still scarce. For further developmental analysis it is important to establish the scale of transcripts variation on a genome-wide level and to identify the whole differentially expressed transcripts, including non-coding transcripts (small RNAs) in three sex stages. By expanding our previously published results about the differential expression patterns of microRNAs in three genders of the rice field eel [29], we sequenced the gonad and brain transcriptomes from three sex stages of rice field eel, respectively. Using stringent filtering criteria and manual curation, we characterize these transcripts in terms of transcript length, conservation and expression pattern during sex reversal and thus anticipate that our catalogue of coding transcripts will enable more developmental and genomic studies directed towards sex reversal mechanisms.

## Methods

### Sample collection and RNA extraction

The rice field eels were obtained from markets located in LiShui Road in Wuhan, China (coordinate N30°27′55.94″, E114°15′33.89″), and were kept in fresh water for seven days before experiment. The rice field eels were anaesthetized with MS222 (350mg/L) before sampling. The sexes were confirmed by microscopic analysis of their gonad sections. A total of 36 adult rice field eels were collected for experiment, 12 individuals for each sexual phase. Tissue samples were collected from the gonad and brain (including pituitary) of different sexual phases of rice field eels (*Monopterus albus*). Total RNA was isolated using Trizol reagent (Invitrogen, USA), suspended in RNase-free water and evaluated by electrophoresis and a Nanodrop 2000c UV-Vis spectrophotometer (Thermo Scientific, Waltham, Mass., USA). The experiments were performed in accordance with the International Guiding Principles for Biomedical Research Involving Animals as promulgated by the Society for the Study of Reproduction. The experimental procedures were also approved by the Institutional Animal Care and Use Committee (IACUC) of Huazhong Agricultural University (Wuhan, China).

### cDNA library construction and transcriptome sequencing

RNA samples (n = 12) from each sexual phase were mixed together by tissue with equivalent amount (3μg RNA per sample) after examination. Sequencing libraries were generated using Illumina TruSeq RNA Sample Preparation Kit (Illumia, San Diego, USA) following manufacturer’s recommendations. Briefly, mRNA was purified from total RNA using poly-T oligo-attached magnetic beads. Fragmentation was carried out using divalent cations under elevated temperature in Illumina proprietary fragmentation buffer. First strand cDNA was synthesized using random oligonucleotides and SuperScript II. Second strand cDNA synthesis was subsequently performed using DNA Polymerase I and RNase H. After adenylation of 3’ ends of DNA fragments, Illumina PE adapter oligonucleotides were ligated to prepare for hybridization. In order to select cDNA fragments of preferentially 200 bp in length, the library fragments were purified with AMPure XP system (Beckman Coulter, Beverly, USA). DNA fragments with ligated adaptor molecules on both ends were selectively enriched using Illumina PCR Primer Cocktail in a 10 cycle PCR reaction. Products were purified (AMPure XP system) and quantified using the Agilent high sensitivity DNA assay on the Agilent Bioanalyzer 2100 system. After cluster generation, the library preparations were sequenced on an Illumina Hiseq 2000 platform and 90 bp paired-end reads were generated.

### Data filtering, reads assembly, and annotation

Raw data were firstly processed to generate clean reads before assembly. In this step, reads with adapters, reads containing over 10% ploy-N and low quality reads were discarded. All the downstream analyses were based on the clean reads. The high quality reads were assembled into unigenes using Trinity with *min_kmer_cov* as 2 and other parameters as default settings [30]. Unigenes were firstly compared by Blastx (E-value<10^−5^) to databases of Nr, Nt and Swiss-Prot, retrieving proteins with the highest sequence similarity with the given unigenes along with their protein functional annotations, and searched against the Pfam database [31] using the HMMER 3.0 package [32] for homologs of protein sequences. The best aligning results were defined according to the feedback E-value, and the first hits were used for further analysis. Functional annotation by Gene Ontology (GO) terms was analyzed by Blast2Go software [33], and summarized using WEGO software[34]. Unigenes were also aligned to the Eukaryotic orthologous groups (KOG) database to predict and classify possible functions. Pathway annotation was performed by blast against the Kyoto Encyclopedia of Genes and Genomes (KEGG) database.

### Differential expression analysis

Prior to differential gene expression analysis, for each sequenced library, the read counts were adjusted by edgeR program package through one scaling normalized factor [35]. To gain the statistic confirmation of the differences in gene expression in the brain and gonad among different sex stages of the rice field eel, we first mapped the reads of each sample to the reference transcriptome using the RSEM software [36] with default settings, then the number of reads that mapped to each unigene was calculated according to the mapping results. Different sequencing samples data were normalized in RPKM (reads per kilo bases per million mapped reads). Differential expression analysis of two sexual phases was performed using the DEGseq (2010) R package, a free R package used to identify differentially expressed genes from RNA-seq data. pvalue was adjusted using qvalue [37]. qvalue<0.005 & |log2 (foldchange)|>1 was set as the threshold for significantly differential expression.

### Screening for most promising sex reversal-related genes

For the selection of maximum differential expression unigene in gonad, the RPKM of each unigene were used in different gonad stage of the rice field eel. While referring to the functional annotation in Nr, Nt, Swiss-Prot and other databases, the candidate genes related to sex differentiation were screened. The screening method was as follows: 1) The RPKM of each unigene among different sequencing samples was |log2 (fold change)|>3. The candidate genes involving gonad growth, development and differentiation were selected referring to gene annotation in Nr, Nt, Swiss-Prot and other database. 2) According to the KEGG pathway classification, a bunch of genes were screened in maximum differences metabolic pathways at different gonadal development stage of eel’s brain and gonad. The candidate genes were selected referring to the gene functional annotation. 3) The unigenes were screened as alternative gene, which had significant expression difference among different sequencing samples and unmatch to any functional annotation databases. The RPKM of each unigene among different sequencing samples was |log2 (fold change)|>10.

### Validation by quantitative real-time PCR (qPCR)

Total RNA was extracted from gonad in different development stage of rice field eel (n = 4) using Total RNA kit II (Omega bio-tek) according to manufacturer's instructions. Total RNA concentrations were measured at 260 and 280 nm using a Nanodrop 2000c UV-Vis spectrophotometer (Thermo Scientific, Waltham, Mass., USA). A 260 nm reading was used to determine the concentration of total RNA and the quality was verified by measuring the 260/280 and 260/230 ratios. Total RNA (1.5 μg) was reverse-transcribed to cDNA using a HiScript II Q RT SuperMix for qPCR with gDNA wiper kit (Vazyme). Primer sequences for cloning and qPCR were designed and purchased from Integrated DNA Technologies (Tianyi Huiyuan, Wuhan, China) and PCR product sizes are listed in S1 Table.

Quantitative real-time RT–PCR experiments were performed in a final volume of 20μL containing 1μL of original cDNA, 0.8μL of each 10 mM primer, 0.4 μL 50× Rox reference, 7.8 μL of RNase and DNase free H_2_O, and 10μL of SYBR Green Master Mix (AceQ qPCR; Vazyme). The protocol was: 7 min at 95°C, followed by 40 cycles of 95°C for 10s, 60°C for 30s, and 72°C for 30s. qPCR was performed in triplicate using Rotor-Gene Q (Qiagen). A melting curve was generated for each run to confirm the specificity of the each product. Fold change was calculated by normalized Ct values against *rpl17* [38] gene using the 2^-ΔΔct^ method[39].

### Statistical analysis

All statistical analyses were performed using Excel 2013 and SPSS 22.0. Each Ct value used for the calculation was the mean obtained from each cDNA in triplicate. The data were tested for statistical differences using a one-way analysis of variances (ANOVA) followed by post-hoc least significance difference (LSD). Unless otherwise noted, values presented are means ± standard error (SE). In all cases, p < 0.05 was considered statistically significant.

### *In situ* hybridization

Foxn 5 mRNA in the gonad were detected by *in situ* hybridisation (ISH). The probe (antisense strand) was transcribed with sp6 RNA polymerase (Roche, Germany), and the negative control was performed using a sence probe. The digoxigenin label was incorporated into the single-stranded probe using the Roche DIG RNA labeling kit. ISH staining was photographed using the confocal Laser-scanning microscope (Leica TCS SP, Argon-Krypton Laser).

## Results

### High throughput sequencing and reads assembly

Raw data have been deposited to NCBI SRA database (accession number: SRX1007627). For gonad, we obtained approximately 32 million raw reads from female samples, 27 million from intersex and 34 million from male samples. For brain, about 35 million raw reads were obtained from female samples, 38 million from intersex and 33 million from male samples. The total amount of raw reads was over 208 million. After filtering low quality reads, reads containing over 10% unidentified bases and reads with adapters, about 200 million clean reads were left, covering 39.94Gb nucleotides. The average Q20 percentage and GC content was 97.97% and 48.34%, respectively. With paired-end joining and gap filling, these reads from six samples were assembled, producing 195,301 unigenes with an N50 of 1505 nt and a mean length of 765 nt, including 16121 unigenes with the length over 2000 nt (Fig A in S1 File).

### Gene annotation and functional classification

**Fig 1:**
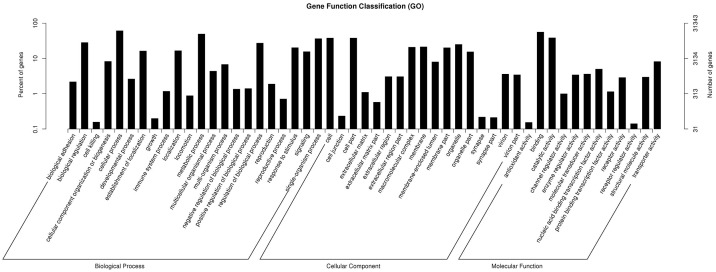
Gene Ontology classifications of assembled non-redundant unigenes.

As a result, 31343 unigenes were annotated in NR and Pfam database. We also blasted the unigenes to the NCBI nucleotide sequences (NT), Swiss-Prot and to KOG database. Together, 44,445 unigenes were annotated at least in one database, among which 8,335 unigenes were annotated in all databases. The Gene Ontology (GO) assignment system were used to classify the functions of the genes annotated based on the NR and Pfam database. The 31343 unigenes were categorized into 54 functional groups based on sequence homology, and were then assigned to three main categories: biological process, cellular component and molecular function, with 20,639 (65.85%), 12,170 (38.83%) and 17,400 (55.51%) unigenes, respectively (Fig 1). A subset of genes was assigned to more than one category according to their multiple functions. In each category, there were dominant groups of genes that involved in the basic biological process, such as the ‘cellular process’, ‘cell part’ and ‘binding’ group, but much fewer genes can be found in the groups such as ‘cell killing’, ‘antioxidant activity’ and ‘receptor regulator activity’.

**Fig 2:**
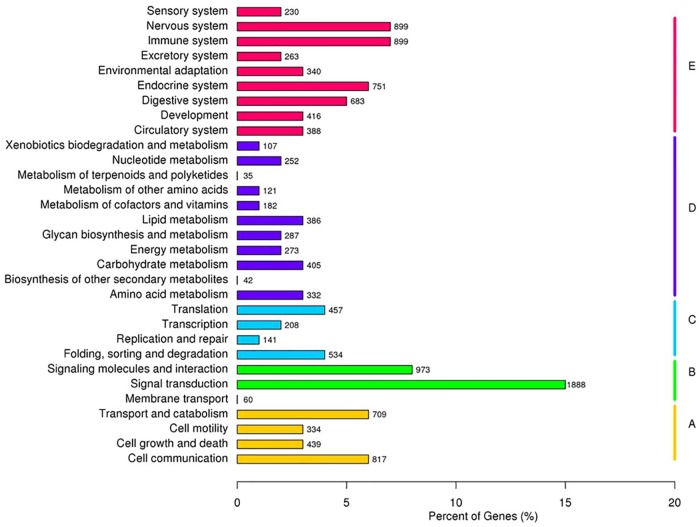
KEGG pathway classification of non-redundant unigenes. **A**: Cellular Processes; **B**: Environmental Information Processing; **C**: Genetic Information Processing; **D**: Metabolism; **E**: Organismal Systems.

Based on the BLASTx results against the KEGG database, we classified the 12609 unigenes into different pathways under each main category except ‘human diseases’, and calculated the number of unigenes under the secondary hierarchy of each pathway. It can be noticed in Fig 2 that the most represented pathway was ‘signal transduction’ under the ‘environmental information processing’ category, containing 1888 unigenes (14.97%), next was ‘signaling molecules and interaction’ (973 unigenes, 7.72%), ‘nervous system’ (7.13%) and ‘immune system’ (7.13%), but much less unigenes were found in ‘metabolism of terpenoids and polyketides’ (35 unigenes, 0.28%), ‘biosynthesis of other secondary metabolites’ (42 unigenes, 0.33%) and ‘membrane transport’ (60 unigenes, 0.48%). These annotations provide a valuable resource for investigating the processes, functions and pathways involved in the sex reversal of the rice field eel.

### Changes in gene expression profiles of the brain among different sex stages of the rice field eel

Gene expression was calculated as RPKM and shows a comparable median distribution across sequencing libraries (Fig B in S1 File). Pearson correlation coefficients reveal the similarity within three brain samples (F_brain, I_brain and M_brain) and three gonad samples(F_gonad, I_gonad and M_gonad), respectively. Major changes can be observed between F_gonad and M_gonad, and between I_gonad and M_gonad, while fewer changes could be found between brain samples (Fig B in S1 File). To rule out any bias in correlation arising from low expression values, we filtered the data for a threshold of 1 RPKM in each sample. To investigate how many genes were differentially expressed among the three sex stages, a threshold of qvalue < 0.005 and | log_2_(fold change)| > 1 was used to minimize false positives. The volcano plot showed the relationships of the fold change and qvalue (adjusted pvalue), red plots stand for genes that were significantly differentially expressed between two samples, while blue plots stand for false positives (Fig C in S1 File).

After filtering the false positives results, it showed that 31 genes were differentially expressed between F_brain and I_brain (S2 Table), 17 of which were up-regulated, including genes encoding Corticotropin-releasing factor receptor 1 (comp164609_c3), Somatotropin (comp96240_c0), and genes involved in signal transduction, such as the Voltage-dependent P/Q-type calcium channel subunit alpha (comp164946_c0). Fourteen genes were down-regulated, with the follicle stimulating hormone beta subunit included. The follicle stimulating hormone is secreted by pituitary and showed great importance in early gonad differentiation and follicle development [40–42]. Twenty-five genes were differentially expressed between I_brain and M_brain, including 18 up-regulated genes and 7 down-regulated ones (S3 Table). Thirty-three genes showed different expression level between F_brain and M_brain, with 9 genes down-regulated and 24 up-regulated (S4 Table).

**Fig 3:**
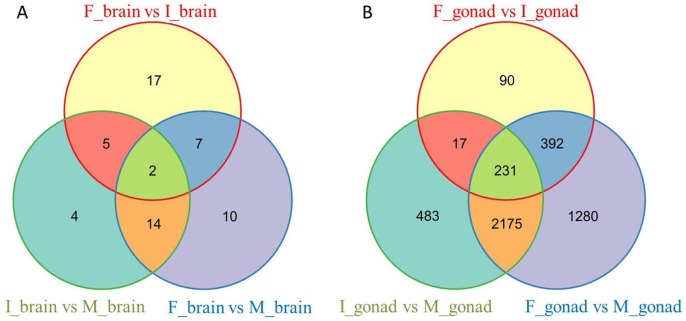
Venn diagram showing differentially expressed genes in brain (A) and gonad tissue (B) from three sex stages. F: female, I: intersex, M: male.

The venn diagram (Fig 3A) indicated that 5 genes were differentially expressed in the intersex comparing to female and male, but showed no changes between female and male, indicating that these genes experienced abundance alteration in the middle of the sex reversal process. One of these genes encodes the luteinizing hormone (comp88640_c0), a very important hormone that can trigger ovulation in females, and meanwhile can stimulate Leydig cells and maintain sperm quality and quantity in males [43–45]. The gene encoding growth hormone together with other 6 genes were found differentially expressed both between female and intersex, and between female and male, but no difference between intersex and male eels. The transcript abundance of two unigenes were found to be significantly changed during the three sex stages, one of which is the immunoglobulin mu heavy chain coding gene (comp164514_c1), suggesting the immune function was affected during the sex alteration. To investigate expression patterns of these genes, we performed unsupervised hierarchical clustering to sex stages and K-means clustering to group genes according to their expression (Figs D and E in S1 File). It showed that many genes are activated at the intersex stage, but still several genes are more strongly induced at the male stage.

### Changes in gene expression profiles of the gonad among different sex stages of the rice field eel

**Fig 4:**
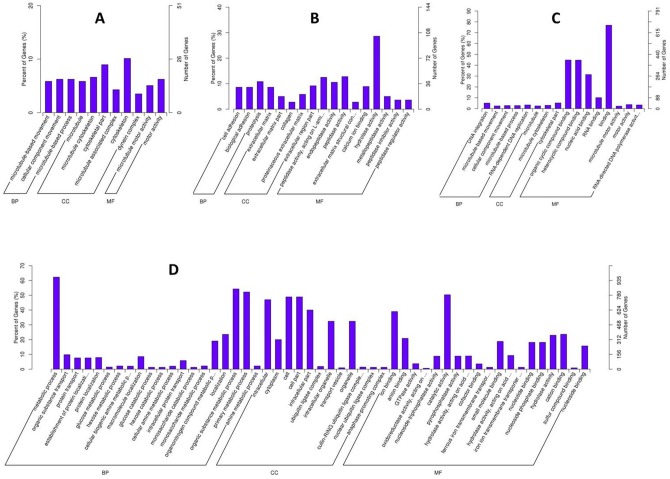
GO classification for differentially expressed genes in gonad between stages. **A**: up-regulated genes comparing F_gonad to I_gonad. **B**: down-regulated genes comparing F_gonad to I_gonad. **C**: up-regulated genes comparing I_gonad to M_gonad. **D**: down-regulated genes comparing I_gonad to M_gonad.

The gonad experienced tremendous morphological changes during sex transformation, along with which a great abundant of genes shifted their expression in different sex stages. To gain insight into the alteration of gene expression in the gonad, multiple comparisons were made between F_gonad, I_gonad and M_gonad. Unlike the brain, far more abundant genes were detected differentially expressed in the gonad between different sex stages. Overall 4668 genes were found to be of unequal abundance in the comparisons, 15.6% of which (730 genes) were detected between F_gonad and I_gonad, 62.3% (2906 genes) between I_gonad and M_gonad, and 87.4% (4078 genes) between F_gonad and M_gonad. To correlate these profiles with gene function, enrichment for GO terms were examined by Blast2Go software [33]. Of the 730 genes with unequal abundance between F_gonad and I_gonad, 322 were up-regulated, which were mainly assigned into 11 sub-categories (Fig 4A). A great abundant of gene were involved in “cellular component” such as cytoskeleton and microtubule complex. Furthermore, several well-known genes that are associated with goand development, such as *spag6* (Sperm-associated antigen 6), *spag17*, *spag1*, *spef2* and *tctex1*, were up-regulated during the transformation from ovary to ovotestis. The other 408 down-regulated genes were assigned into 17 sub-categories, most of which were molecular function related (Fig 4B), involving in the activities of different kind of enzymes. Over a hundred of these genes were hydrolase-activity related. Besides, several gonad development related genes such as vitellogenin receptor, dual specificity testis-specific protein kinase and *sox3*, were also down regulated during the transformation from ovary to ovotestis. Pathway enrichment analysis revealed these down-regulated genes were mainly enriched in PI3K-Akt signaling pathway (28 genes, qvalue<0.01), protein digestion and absorption (26 genes, qvalue<0.01), focal adhesion (25 genes, qvalue<0.01) and ECM-receptor interaction (24 genes, qvalue<0.01, Fig F in S1 File).

**Fig 5:**
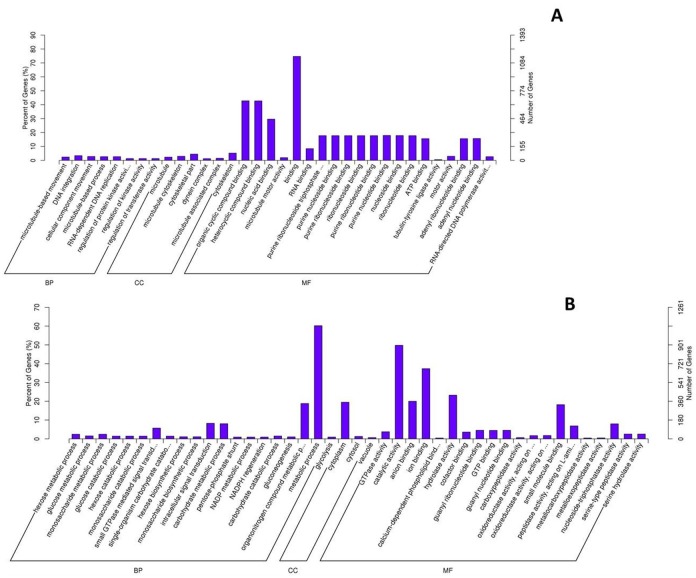
GO classification for differentially expressed genes in gonad between female and male stage. **A**: up-regulated genes. **B**: down-regulated genes.

Of the 2906 genes differentially expressed between I_gonad and M_gonad, 1064 were up regulated and were categorized into 16 different sub-categories (Fig 4C), with the majority of genes related with “binding” functions. These genes were mainly enriched in lysine degradation pathway (qvalue<0.05, Fig G in S1 File). The other 1842 down-regulated genes showed a great enrichment in the “metabolic process”, “cell”, “cell part” and “catalytic activity” (Fig 4D). These genes were mainly enriched in metabolic pathway (141 genes, qvalue<0.05), lysosome (30 genes, qvalue<0.01) and Protein processing in endoplasmic reticulum (32 genes, qvalue<0.05, Fig H in S1 File). Finally, the comparison between F_gonad and M_gonad revealed that over four thousand genes shifted their transcript abundance, with 1940 up-regulated and 2138 down-regulated. It can be noticed that these changes seriously affected the molecular binding process and metabolic process (Fig 5). Those up regulated genes were mainly enriched in Lysine degradation pathway (16 genes, qvalue<0.01, Fig I in S1 File), while those down-regulated ones were mainly enriched in metabolic pathway (166 genes, qvalue<0.05), lysosome (38 genes, qvalue<0.01), biosynthesis of secondary metabolites (55 genes, qvalue<0.05), and microbial metabolism during the transformation (39 genes, qvalue<0.05, Fig J in S1 File).

To investigate expression patterns of these genes, we performed unsupervised hierarchical clustering to sex stages and K-means clustering to group genes according to their expression (Figs D and K in S1 File). These genes were divided into 6 sub clusters based on their expression pattern. Within these genes, 231 were found with different expression in three sex stages (Fig 3B), including several reproduction-related genes, such as sperm flagellar protein 2-like (comp160618_c0), *amh* (comp148436_c0), AMY-1-associating protein (comp146331_c0), Sperm-associated antigen 6 (comp67196_c0) and piwi-like protein 1 (comp164266_c0). All these genes followed a trend that it maintained a low expression level in the female stage and kept rising through intersex to male stage. The piwi-like protein 1 was one of highly expressed genes in the male gonad, it plays a central role during spermatogenesis by repressing transportable elements and preventing their mobilization, which is essential for the germline integrity [46]. The vitelline membrane outer layer protein 1 homolog and *cyp19a1*, along with other 390 genes, were found with different expression level between F_gonad and I_gonad, and between F_gonad and M_gonad, but of the same abundance between I_gonad and M_gonad. The *cyp19a1* encodes a member of the cytochrome P450 superfamily of enzymes, which catalyzes the last steps of estrogen biosynthesis [47–49]. The down-regulation of *cyp19a1* indicated that the synthesis of estrogen was reduced when the rice-field eel stepped into the intersex.

### Screening and validation of the most promising candidate genes affecting the sex reversal in the rice field eel

**Fig 6:**
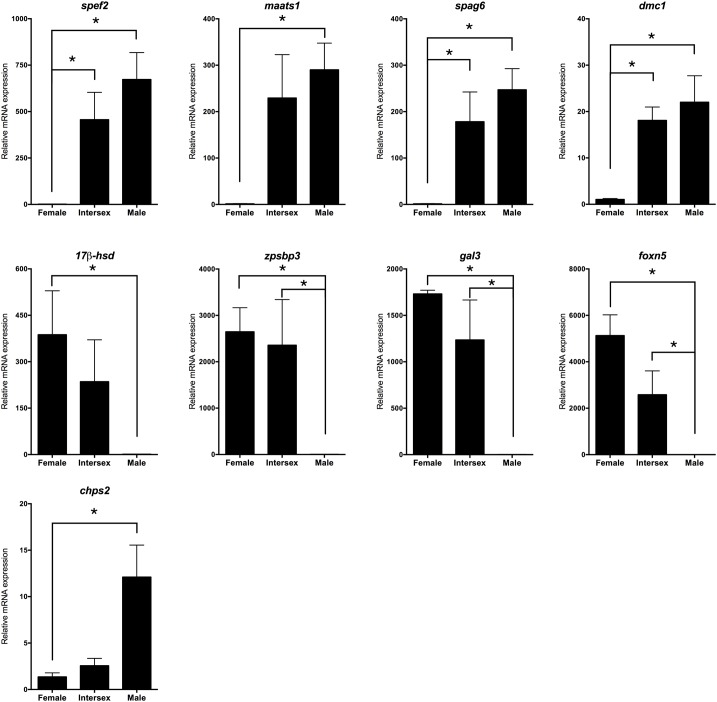
RT-qPCR validation for 9 gonad differentiation related genes. The “*” stands for p < 0.05.

**Fig 7:**
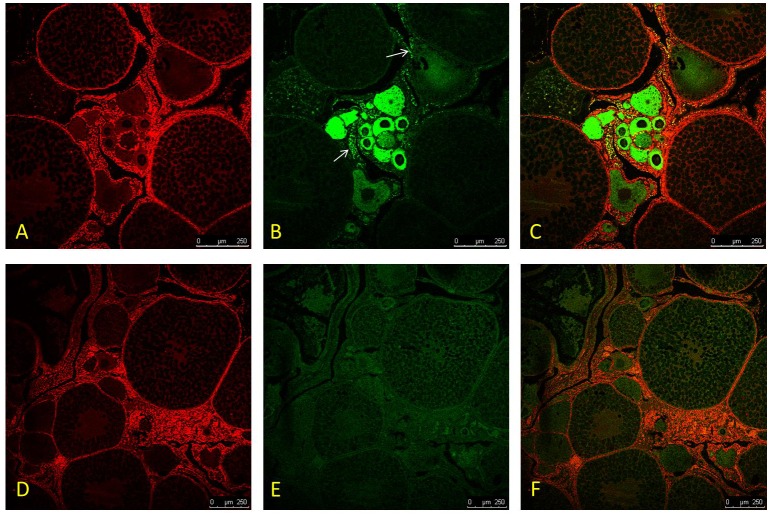
*In situ* hybridization for *foxn5* in female gonad of rice field eel. The digoxigenin label was incorporated into the single-stranded probe, and a sense a sense probe was used as negative control. It showed that the expression of *foxn5* was located in the egg envelope and the early phase of follicles. A: nucleus dyed by PI; B: localization of *foxn5*, marked by white arrow: C: merged A and B. D-F for negative control.

A total of 19 candidate genes that related to gonad differentiation were screened according to the gene annotation and analysis of gene differential expression (Table 1). According to the gene functional annotations, these genes were functionally involved in meiosis, steroid metabolism, sperm biosynthesis, reproduction and gonadal developmental in maintaining important physiological processes. To validate the transcriptome sequencing results, 9 unigenes from these 19 genes were randomly selected to determine their expression levels in different gonads of the rice field eel by RT-qPCR (Fig 6). The expression levels of 8 genes were consistent with the transcriptome data set except for *chps2*. Based on our data set, the expression of *spef2*, *maats1*, *spag6* and *dmc1* were more abundant in males, but was barely detected in females. While the expression of *17β-hsd12*, *zpsbp3*, *gal3* and *foxn5* was mainly detected in ovary. These genes could be the most promising candidates affecting the rice field eel’s sex transformation process. We chose one of these genes, *foxn5*, to perform in situ hybridization analysis. The result showed that the expression of foxn5 was detected mainly in the egg envelope and the early phase of follicles, but not in the mature ones (Fig 7), suggesting the functional importance of foxn5 in the early development of the follicles in the rice field eel.

**Table 1:** Partial candidate unigenes screened from gonad transcriptsm of *M*. *albus*.

Unigene_id	Gene symbol	p_value	Unigene annotation	The species of maximum similarity
comp160752_c1	*zpsbp3*	9.91×10^−104^	zona pellucida sperm-binding protein 3-like	*Larimichthys crocea*
comp73847_c0	*chps2*	8.71×10^−93^	chymotrypsinogen 2	*Sparus aurata*
comp166567_c0		4.55×10^−64^	elastase 4 precursor	*Epinephelus coioides*
comp96181_c0		1.77×10^−39^	histone H2A type 2-B-like	*Oreochromis niloticus*
comp150930_c0	*foxn5*	3.87×10^−38^	forkhead box protein N5-like	*Larimichthys crocea*
comp137762_c1		6.18×10^−36^	trypsinogen 2	*Solea senegalensis*
comp144167_c0	*17β-hsd12*	1.35×10^−32^	17-beta-dehydrogenase 12	*Danio rerio*
comp162627_c1	*dzip1*	3.93×10^−29^	zinc finger protein Dzip1	*Takifugu rubripes*
comp160618_c0	*spef2*	2.26×10^−20^	sperm flagellar protein 2	*Notothenia coriiceps*
comp64143_c0	*dmc1*	1.42×10^−14^	meiotic recombination protein DMC1/LIM15	*Oreochromis niloticus*
comp146331_c0	*maats1*	3.06×10^−14^	MYCBP-associated protein expressed in testis 1-like	*Larimichthys crocea*
comp166662_c0		1.42×10^−13^	differentially regulated trout protein	*Siniperca chuatsi*
comp134575_c0	*gal3*	9.42×10^−13^	galectin-3	*Oreochromis niloticus*
comp127487_c0	*vmo1*	1.58×10^−11^	vitelline membrane outer layer protein 1	*Larimichthys crocea*
comp153639_c0		2.37×10^−10^	calcium-binding protein 5	*Oreochromis niloticus*
comp161819_c0		7.90×10^−10^	germ cell-less protein-like 1	*Oreochromis niloticus*
comp56122_c0		6.13×10^−08^	hypothetical protein LOC100698933	*Oreochromis niloticus*
comp146306_c0		9.43×10^−06^	A disintegrin and metalloproteinase with thrombospondin motifs 13-like	*Oryzias latipes*
comp67196_c0	*spag6*	1.58×10^−05^	Sperm-associated antigen 6	*Larimichthys crocea*

## Discussion

In vertebrates, reproduction is primarily controlled by the HPG (Hypothalamic-Pituitary-Gonadal) axis, and the structure of this endocrine pathway is highly conserved in jawed vertebrates. The hypothalamic neuroendocrine system regulates synthesis and release of the gonadotropins, follicle-stimulating-hormone (FSH) and LH, from the pituitary, which in turn stimulate gonadal development, in particular via the induction of sex steroid synthesis. Sex steroids feedback to the hypothalamus and the pituitary, thereby regulate gonadotropin synthesis and release [50, 51]. So we sequenced the brain (including pituitary) and gonad tissue to explore gene expression patterns among sex stages. In our study, the level of the FSH beta subunit precursor was different between F_brain and I_brain, but with no difference between F_brain and M_brain. To our knowledge this is because that the FSH stimulates the maturation of germ cells in both females and males. In females, the FSH initiates follicular growth, specifically affecting granulosa cells. In males, it is required for the determination of Sertoli cell number, and for induction and maintenance of normal sperm production[52]. The LH beta subunit was differentially expressed comparing F_brain to I_brain, and I_brain to M_brain, but of the same level between F_brain and M_brain. This is probably because in both females and males, the LH is essential for reproduction. Beside the function of triggering ovulation, the LH can also stimulate Leydig cells and maintain sperm quality and quantity in males [43–45], and thus it was also call interstitial cell-stimulating hormone (ICSH) [44]. The growth hormone (GH, comp96240_c0) show a higher expression level in M_brain than in F_brain, this could be one of the reasons that the male eels grow faster and larger than females [53, 54].

The gonad experienced tremendous morphological changes during sex transformation, and far more genes shifted their expression within this process. Several genes involved in the PI3K-Akt signaling pathway decreased their expression during the ovary-to-ovotestis transformation, such as serine/threonine-protein kinase Sgk1-like (comp154545_c0), cyclic AMP-responsive element-binding protein 1 (comp163815_c2) and DNA-damage-inducible transcript 4 protein (comp145559_c0). The PI3K-Akt signaling pathway regulates fundamental cellular functions such as transcription, translation, proliferation, growth and oocyte maturation in fish [55, 56], and can be activated by estrogenic hormone and the autocrine/paracrine system [56–58], thus we speculated that one of the reasons that caused the down-regulation of this signaling pathway might be changes of receptors’ activity on the cell membrane, the lack of estrogenic hormone and the weak ovarian autocrine/paracrine activity. The synthesis of estrogenic hormone was strongly regulated by the cytochrome p450 aromatase and the 17 beta-hydroxysteroid dehydrogenase. The cytochrome p450 aromatase (cyp19) is the terminal enzyme is the steroidogenic pathway that converts androgens into estrogens, therefore, appropriate expression of the enzyme is of great importance for reproduction as well as being pivotal in sex differentiation for most vertebrates. In our data, the cyp19 was expressed mainly in females, while in males it was barely detected, which was in accordance with other researchers’ experimental results [59]. It is well known to us that microRNA is playing a crucial role in modulating gene expression, and it has become clear that cyp19a was one of the targets of miR-181 and let-7f [60, 61]. Thus the expression of these two miRNAs should theoretically be lower in females than in males, since miRNAs can only down regulate target genes. However, according to our previous comparative study of rice field eel microRNA transcriptomes, the expression of let-7f maintained a high but equal level within three genders, while that of the miR-181 was found more abundant in females comparing to males [29]. This is probably because of the multi-functions of each miRNA, and diverse regulating pathway of genes. The 17beta-hydroxysteroid dehydrogenase 12 (17β-Hsd12) is an important enzyme that can regulate the biological activity of steroid hormones at the pre-receptor level in many microorganisms, invertebrates and vertebrates [62]. It mainly catalyzes the reduction of C17-ketosteroids to their corresponding hydroxylated forms as well as the reverse reaction, and is able to convert inactive or less active steroid hormones into more potent ones and vice versa, certain 17β-Hsds play a key role, especially in the regulation of estrogen and androgen levels [62–64]. In rice field eel, the expression of *17β-hsd12* was stage-specific, which was more abundant in females while barely detected in males according to our data set, and was supported by qPCR.

Genes related to lysosome pathway were kept down-regulated from ovary through ovotestis to testis according to our dataset. This probably because that lysosome plays a key role in the development of fish oocyte [65], and this down-regulation might be one of the main reasons that caused ovary degeneration.

We screened out 19 candidate genes that could be most related genes to the sex reversal process, and part of their expression were verified by qPCR. Such as, the *dmc1* is required for homologous chromosome synapsis during meiosis in both mammals and fishes [66, 67], while the *spef2* and *spag6* were both found crucial to spermatogenesis and flagellar assembly [68, 69]. The *17β-hsd12*, a member of the hydroxysteroid (17β) dehydrogenase superfamily which could affect the reproductive system by involving the metabolism of sexual hormones, was found playing an important role in ovarian function, meiosis and regulation of fertility[70]. Although several of these candidate genes were not deeply studied in fish yet, their roles in the sex reversal process could not be neglected according to their expression patterns. Besides this, it is worth noticed that although there were thousands of genes with unequal expression patterns through sex stages, most of them were not (directly) related with the sex determination or gonad development process, and some were with considerably high expression level. Those genes involved in basal metabolism could also be of great importance during sex transformation. Genes with crucial function and gonad development involved, however, usually maintained a relatively low abundance. Thus there should be more pathways that could affect the sex reversal process, and further comprehensive studies were needed.

### Conclusion

We provide a comprehensive study of the rice field eel transcriptome of brain and gonad tissue in three sex stages. Our data produced over 195 thousand unigenes and 44,445 were annotated. Comparative study between stages provided multiple differentially expression genes in brain and gonad tissue, which could be candidates involved in sex reversal. Gene expression profiling reveals the relationship between enriched pathways and sex stages. Our study provides a rich developmentally relevant resource, integration of which will enable new genomic and genetic studies in the near future.

## Supporting information

S1 Table Primers used in this study for validalation of unigene by RT-qPCR. (XLSX)

S2 Table Genes differentially expressed between F_brain and I_brain. (XLSX)

S3 Table Genes differentially expressed between M_brain and I_brain. (XLSX)

S4 Table Genes differentially expressed between F_brain and M_brain. (XLSX)

S1 File Supporting data analysis figures. (PDF)
