# Supplementary material for: Genome-wide analysis of brain and gonad transcripts reveals changes of key sex reversal-related genes expression and signaling pathways in three stages of Monopterus albus
Source: PLoS One. 2017 Mar 20;12(3):e0173974. doi: 10.1371/journal.pone.0173974 (PMC5358790; doi:10.1371/journal.pone.0173974)
Supplement: S1 File — (PDF) [file pone.0173974.s005.pdf]

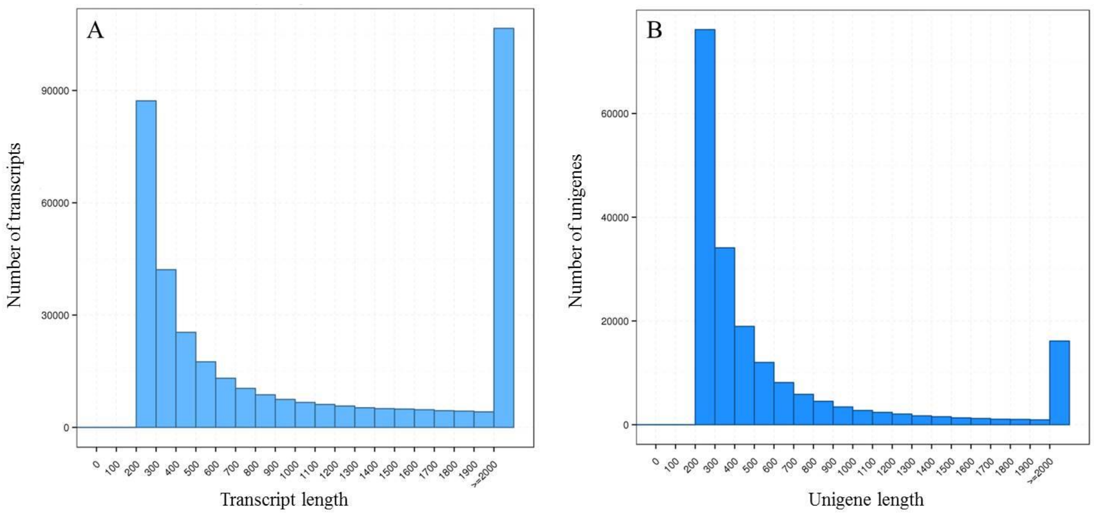

Figure A. Length distribution of transcripts and unigenes. **A**: transcript length distribution; **B**: unigene length distribution.

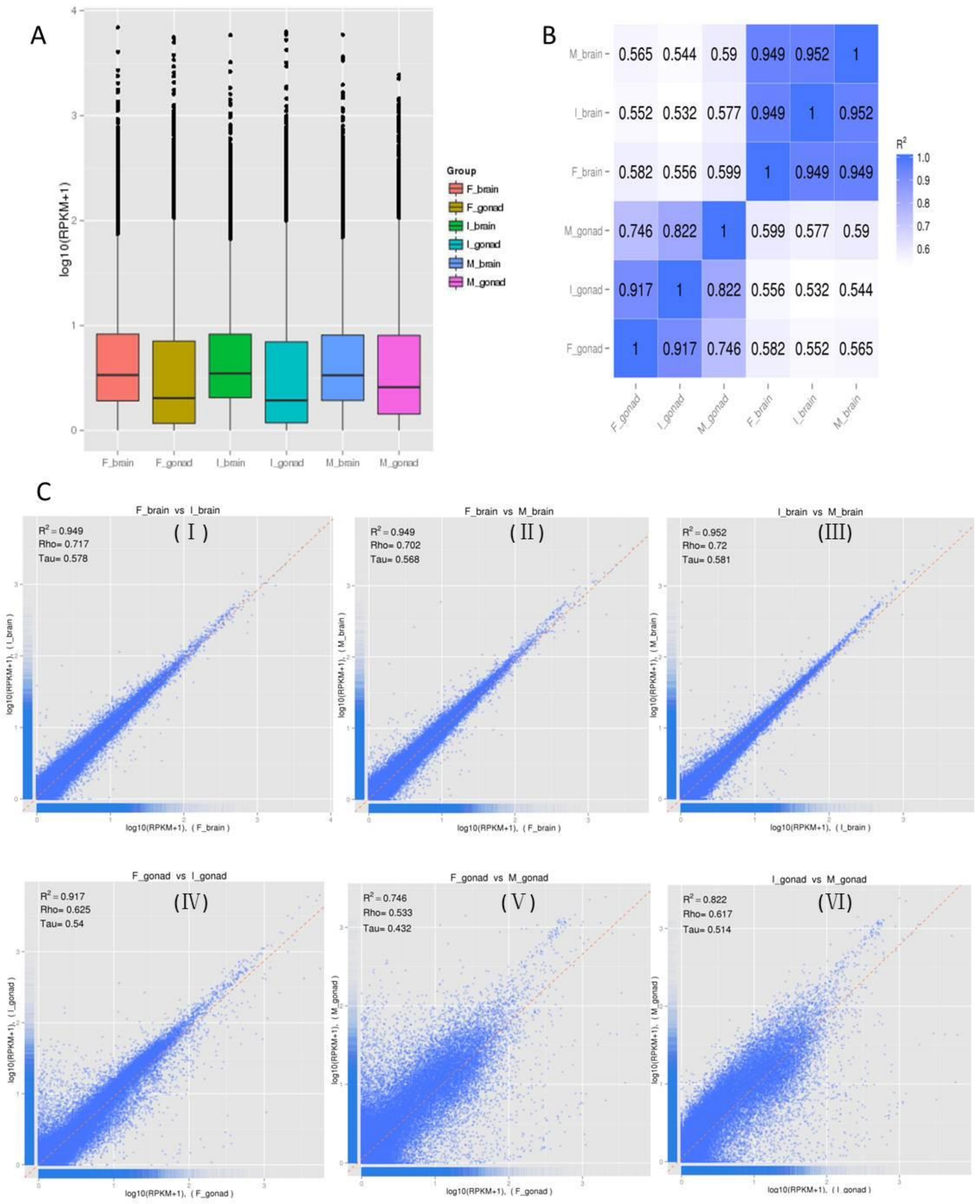

Figure B. Generation of RNA-sequencing libraries. **A**: RPKM distribution across six samples; **B**: Heat map to show Pearson correlation of expression (RPKM) between all 6 RNA-seq libraries; **C**: Scatter plots to show stage specific Pearson correlation between RNA-seq data.

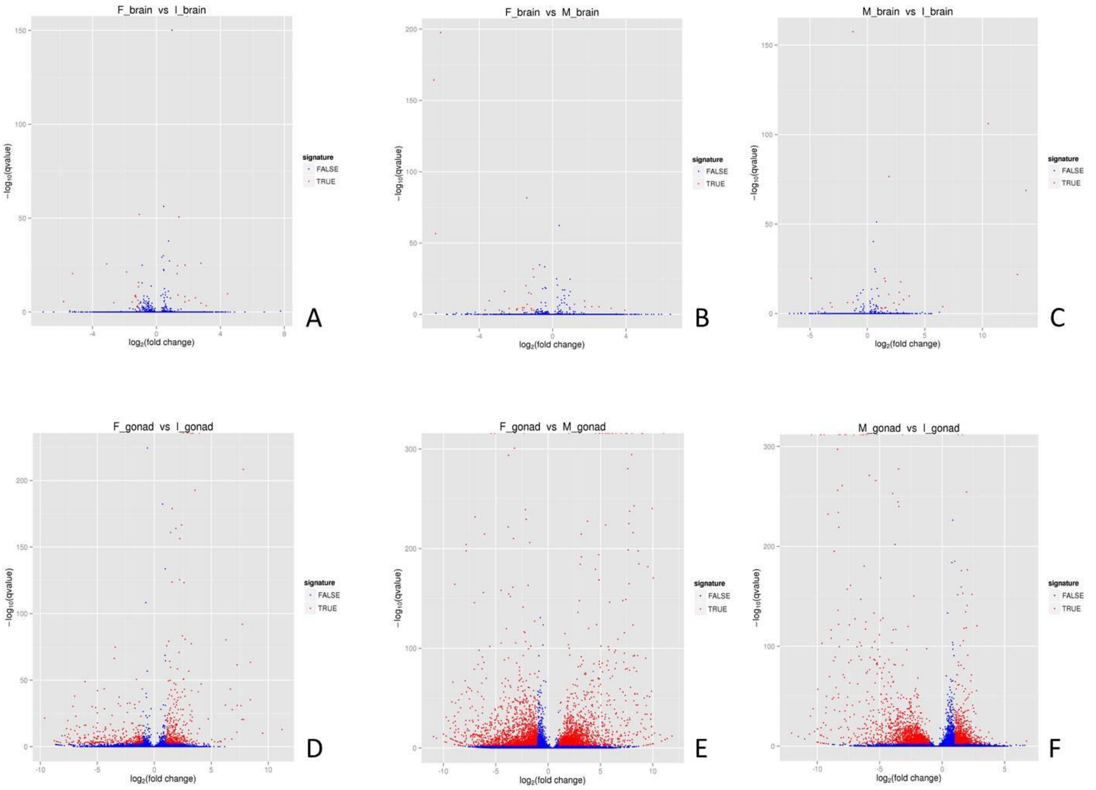

Figure C. Volcano plot displaying differential expressed genes between sex stages. A, B and C for brain tissue, D, E and F for gonad tissue.

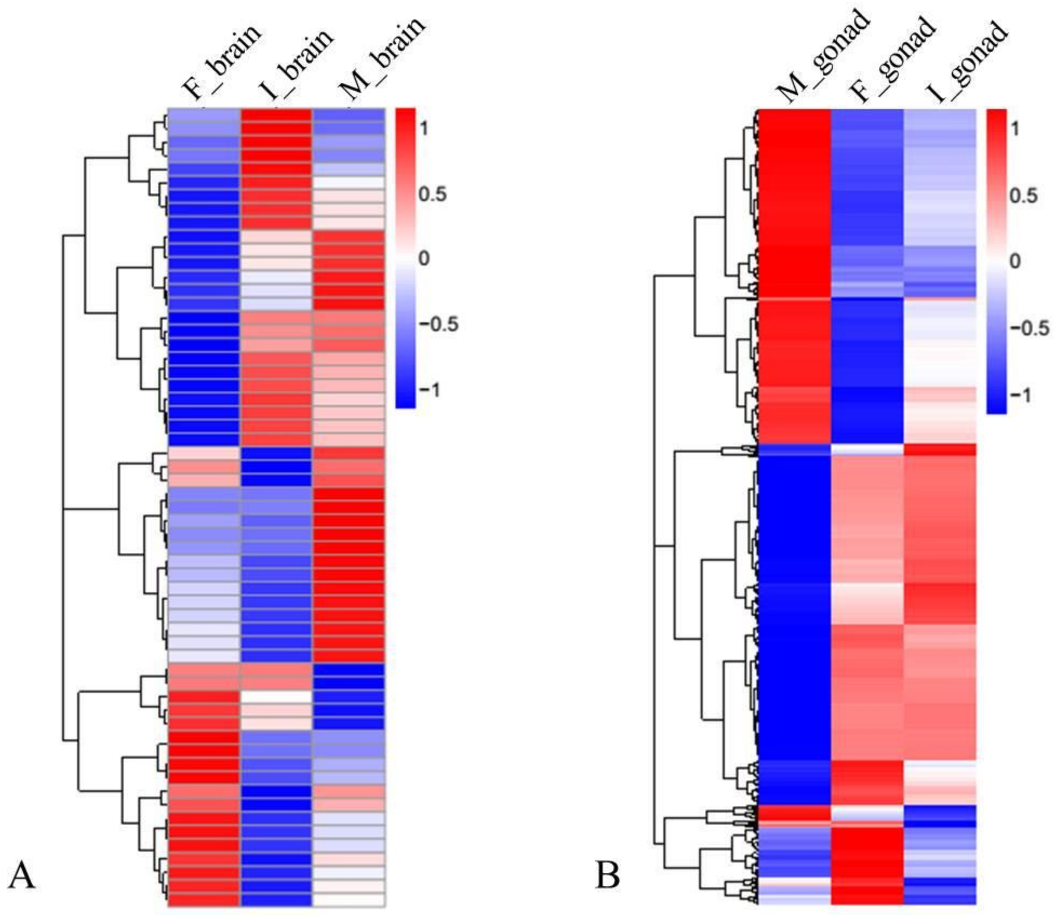

Figure D. Clustering analysis of differential gene expression pattern in brain (**A**) and gonad tissue (**B**) from three sex stages. F: female, I: intersex, M: male.

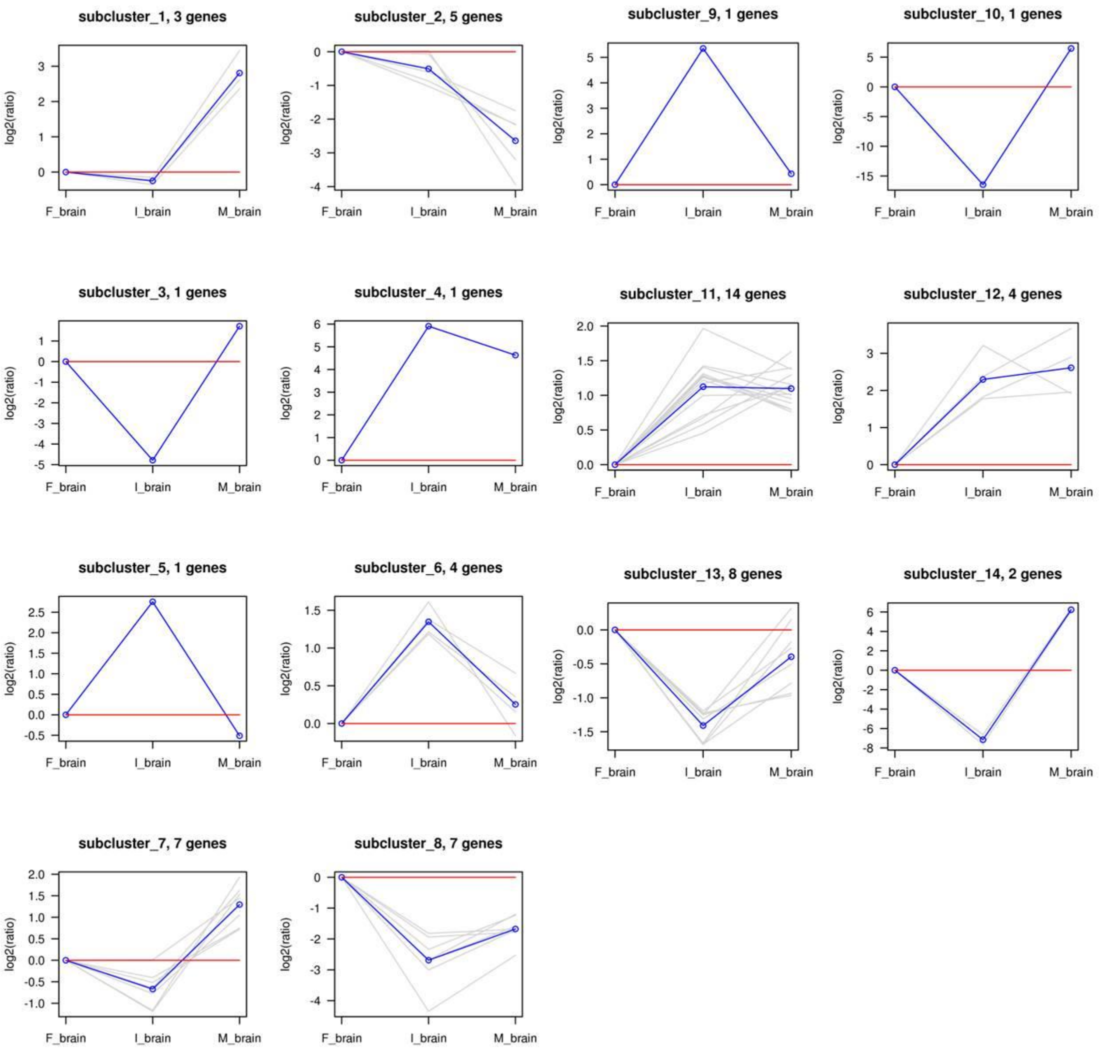

Figure E. Grouping genes according to their expression in brain by K-means clustering.

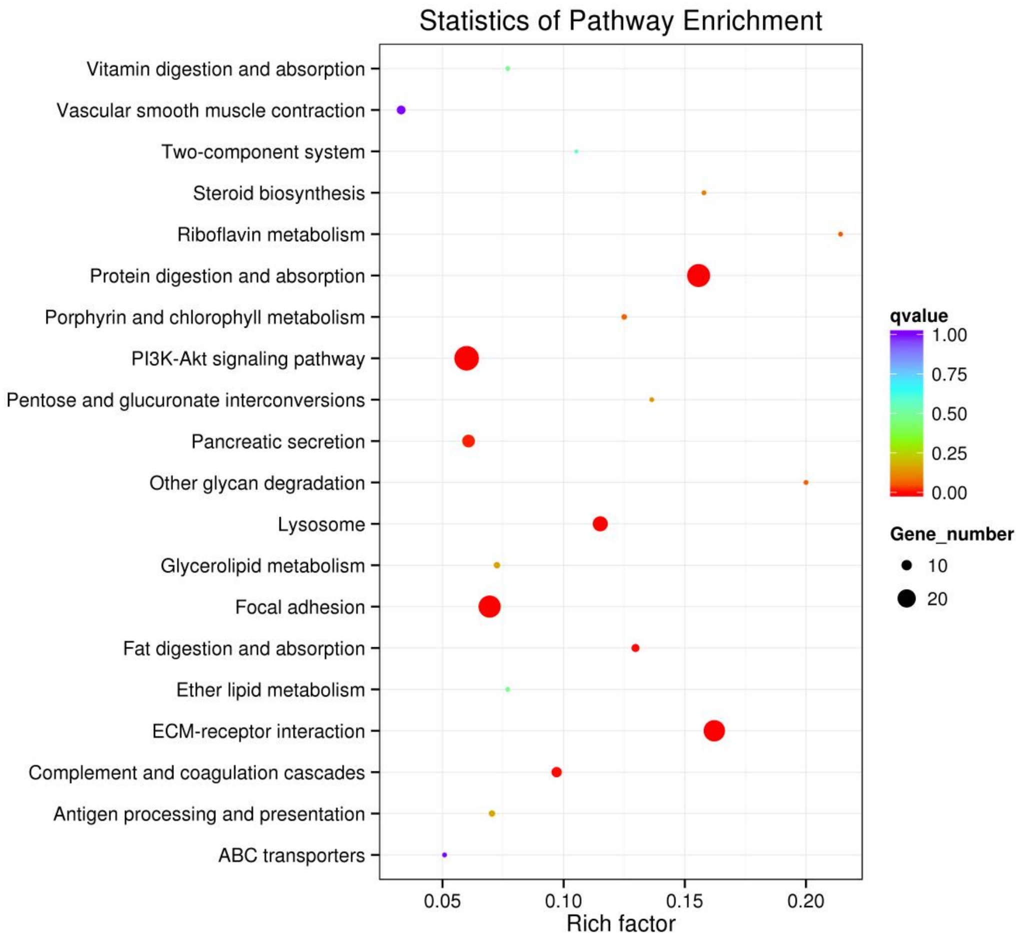

Figure F. Signaling pathway enrichment for the down-regulated genes between F_gonad and I_gonad.

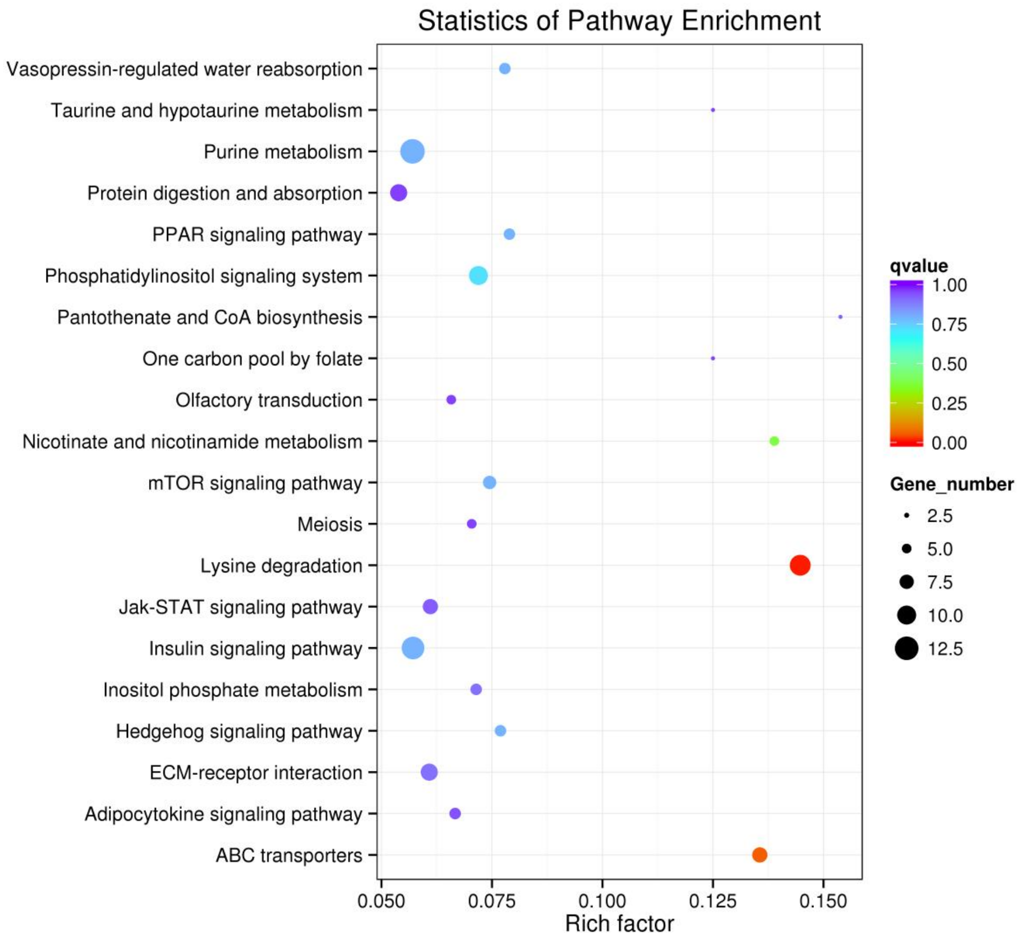

Figure G. Signaling pathway enrichment for the up-regulated genes between I_gonad and M_gonad.

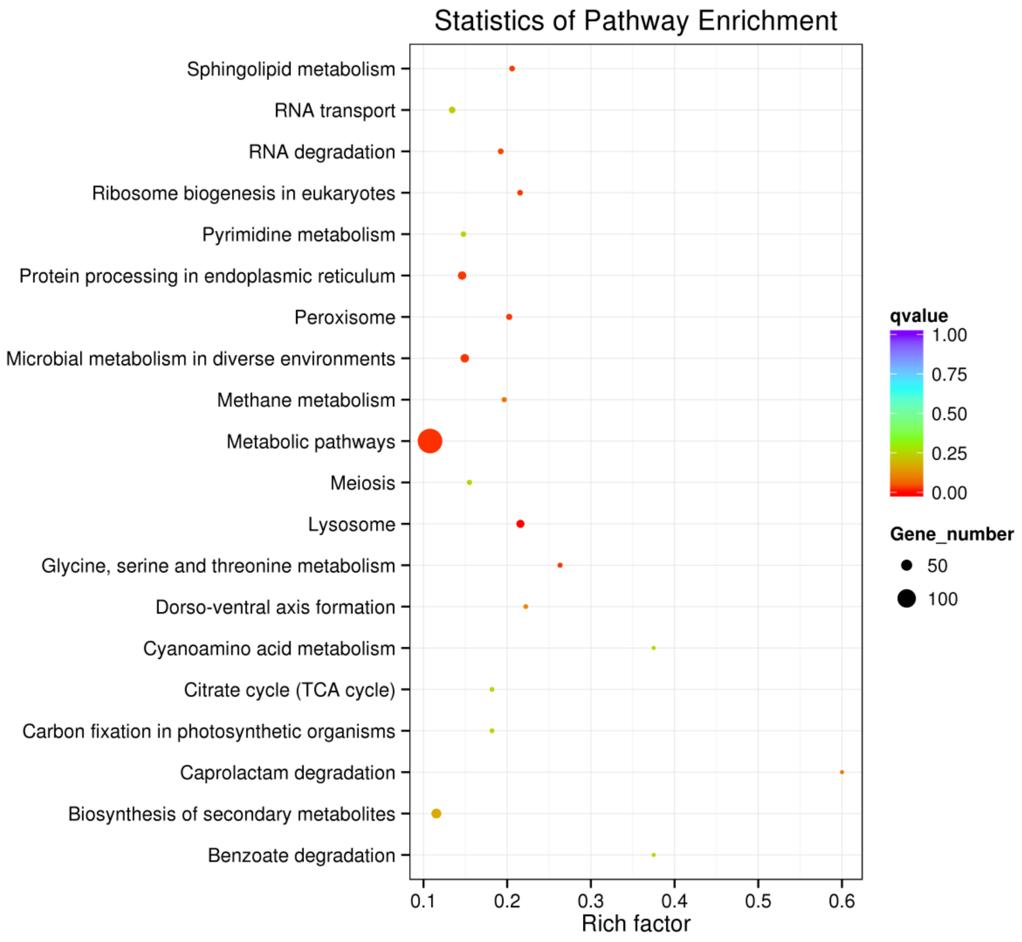

Figure H. Signaling pathway enrichment for the down-regulated genes between I_gonad and M_gonad.

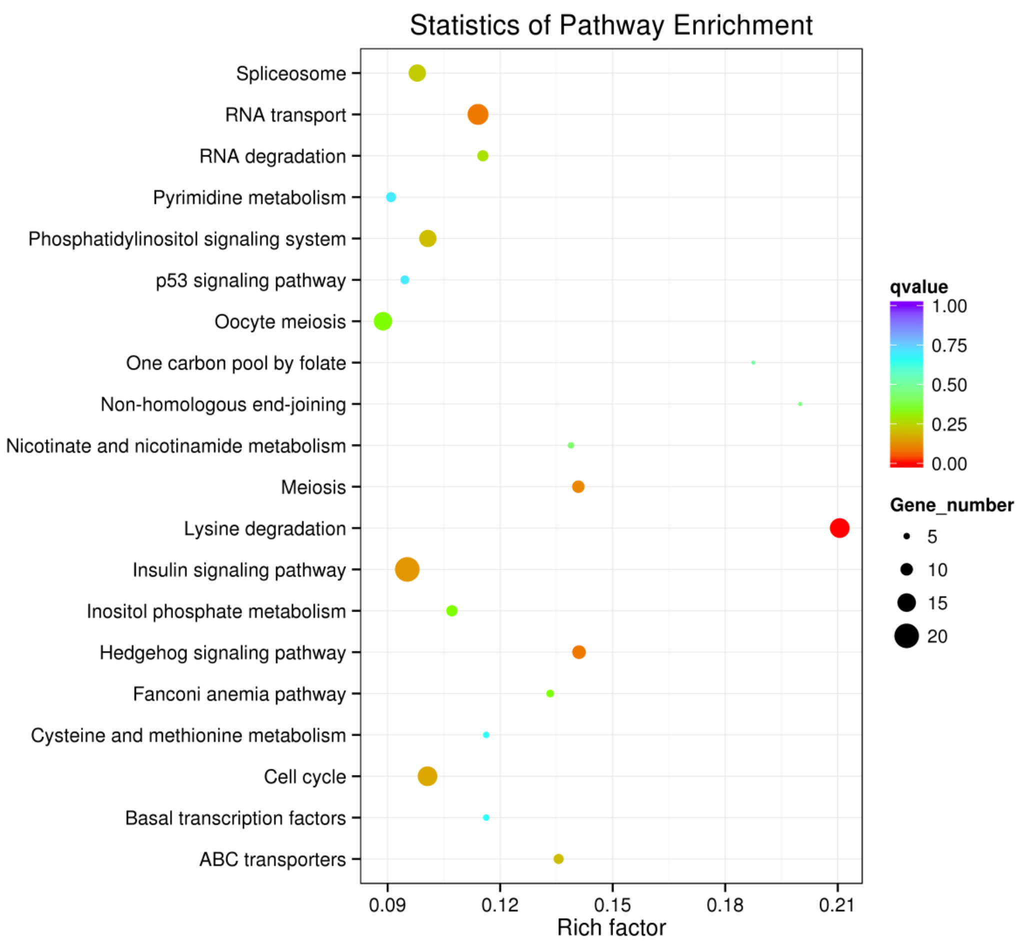

Figure I. Signaling pathway enrichment for the up-regulated genes between F_gonad and M_gonad.

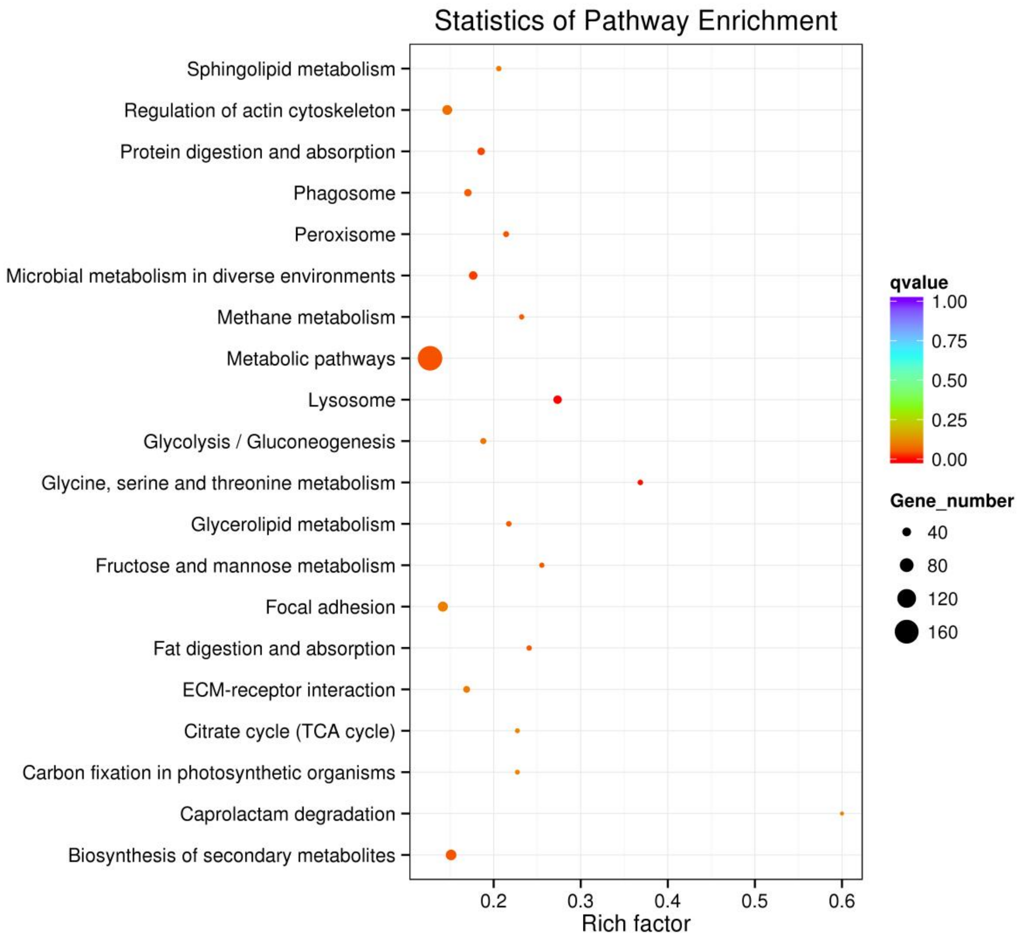

Figure J. Signaling pathway enrichment for the down-regulated genes between F_gonad and M_gonad.

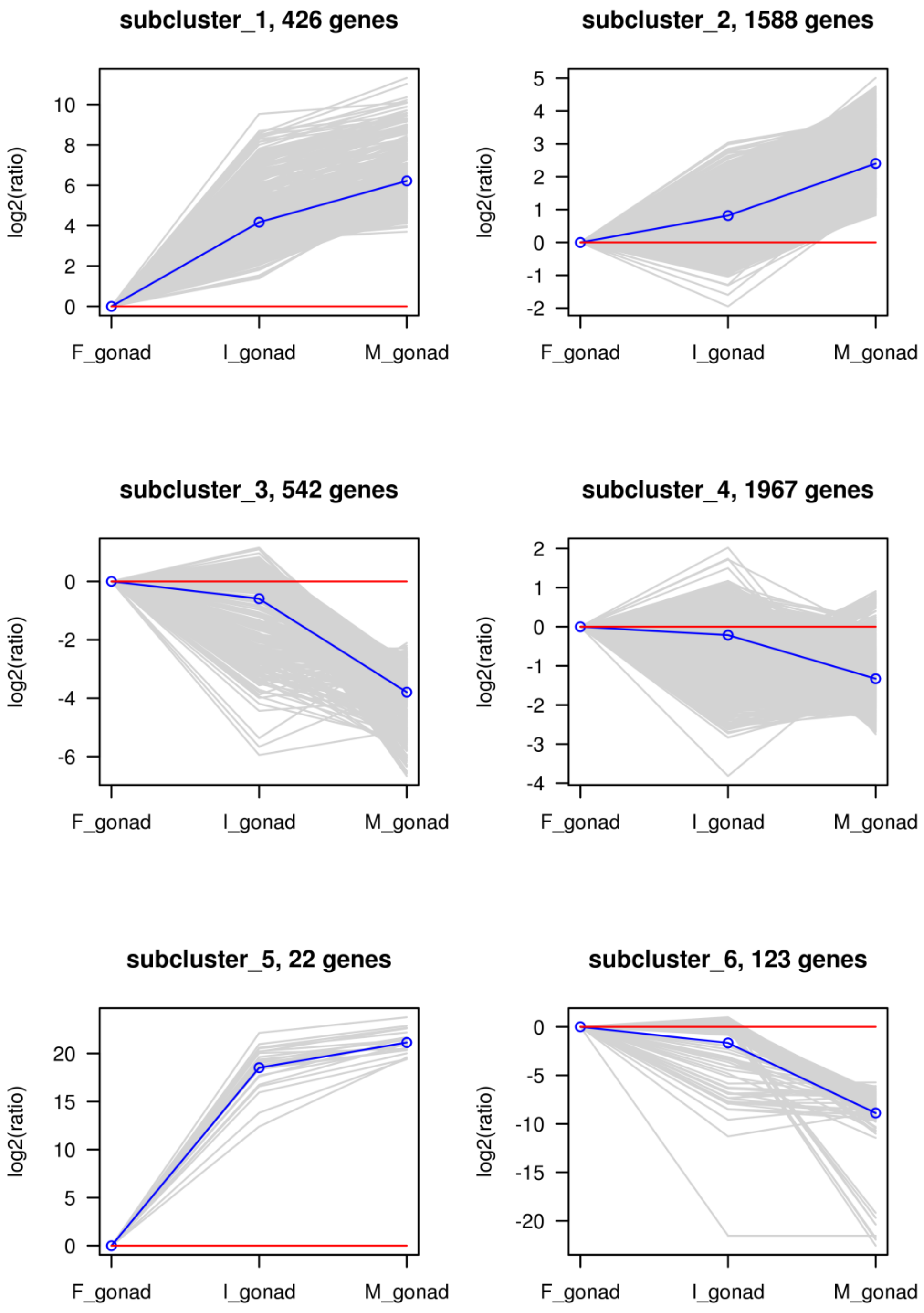

Figure K. Grouping genes according to their expression in gonad by K-means clustering.
